# A Recent Development of a Network Approach to Assessment Data: Latent Space Item Response Modeling for Intelligence Studies

**DOI:** 10.3390/jintelligence12040038

**Published:** 2024-03-28

**Authors:** Inhan Kang, Minjeong Jeon

**Affiliations:** 1 Department of Psychology, College of Liberal Arts, Yonsei University, Seoul 03722, Republic of Korea; qpsy@yonsei.ac.kr; 2 Social Research Methodology, Department of Education, School of Education and Information Studies, University of California, Los Angeles, CA 90095, USA

**Keywords:** intelligence, measurement, item response theory, latent space, conditional dependence

## Abstract

This article aims to provide an overview of the potential advantages and utilities of the recently proposed Latent Space Item Response Model (LSIRM) in the context of intelligence studies. The LSIRM integrates the traditional Rasch IRT model for psychometric data with the latent space model for network data. The model has person-wise latent abilities and item difficulty parameters, capturing the main person and item effects, akin to the Rasch model. However, it additionally assumes that persons and items can be mapped onto the same metric space called a latent space and distances between persons and items represent further decreases in response accuracy uncaptured by the main model parameters. In this way, the model can account for conditional dependence or interactions between persons and items unexplained by the Rasch model. With two empirical datasets, we illustrate that (1) the latent space can provide information on respondents and items that cannot be captured by the Rasch model, (2) the LSIRM can quantify and visualize potential between-person variations in item difficulty, (3) latent dimensions/clusters of persons and items can be detected or extracted based on their latent positions on the map, and (4) personalized feedback can be generated from person-item distances. We conclude with discussions related to the latent space modeling integrated with other psychometric models and potential future directions.

## 1. Introduction

The study of intelligence began with the development of measurement tools, such as intelligence test items (e.g., Binet-Simon test invented in 1904; Binet and Simon 1948) and psychometric models (Spearman 1904). For research on intelligence, it is essential, beyond creating test items, to analytically inspect what aspect in intelligence these items measure and how accurately and stably they measure it. In modern intelligence research, this process has been concretized through psychometric and statistical methodologies, particularly item response theory (IRT) models.

Traditional psychometric models for intelligence research assume **conditional independence (CI)**. Typically, model parameters pertain to either respondents or test items. From item response data, these models capture and separate person and item effects. For example, the Rasch model (one-parameter IRT model; Rasch 1961) is expressed by the following equation:

$$logit\left(P\left(Y_{pi}=1|\theta_{p},b_{i},\xi_{p},\zeta_{i}\right)\right)=\theta_{p}+b_{i}.$$

Here, $\theta_{p}\in R$ represents the general ability (e.g., intelligence) of person *p* (p=1,⋯,P), and $b_{i}\in R$ represents the characteristic (e.g., difficulty/easiness) of item *i* (i=1,⋯,I). Through these parameters, the Rasch model estimates the main effects of individuals and items on response accuracy. In addition, the model implicitly assumes that, when both effects are controlled for, there is no correlation between item responses. This CI assumption, shared by most fundamental latent variable models, means that $\theta_{p}$ and $b_{i}$ can perfectly account for a correlational structure of item responses.

From a practical point of view, CI is related to **unexplained interactions** between persons and items. CI in the Rasch model only considers the main effects (captured by $\theta_{p}$ and $b_{i}$) without accounting for potential person-item interactions. The absence of interactions implies that, for example, all respondents in a test perceive the difficulty of an item in the same way. In other words, item difficulty is a global property that does not vary across persons. This also suggests that the latent ability level of one person is exerted uniformly across all items. However, in actual intelligence tests, educational assessments, psychological tests, etc., perceived item difficulty can vary across persons due to, not only test-intrinsic reasons such as declines in motivation/concentration and sequential effects, but also test-extrinsic reasons such as cultural background, upbringing, educational level, subpopulation differences, etc. (Bolsinova et al. 2017b; Jeon et al. 2021; Kang et al. 2023). This indicates the presence of interaction effects between persons and items, leading to the occurrence and observation of **conditional dependence (CD)**. Some traditional IRT models also include terms representing such interaction effects. For instance, the item discrimination parameter $a_{i}$ can be implemented into Equation (1) as a coefficient to $\theta_{p}$, which yields the two-parameter logistic IRT model (2PLM). In this case, the model has the product term $a_{i}\cdot \theta_{p}$, which captures person-item interaction effects in a systematic way as a combination of the main person and item effects (similar to a moderation/interaction effect in regression models). However, actual interactions may not be fully expressed by these main effect terms, and previous studies on CD acknowledged that unexplained interactions and CD may persist even with the product term implemented in a measurement model (e.g., Kang et al. 2022b, 2023).

CD that can occur in intelligence tests is not just undesirable residual variations or violations of model assumptions, but a data-based source of valuable information on respondents and items. If respondents perceive item difficulties differently, items that a specific individual finds relatively easier or more challenging can be identified. Utilizing this information on person-item interactions, more precise measurements of intelligence can be achieved; further, personalized and specialized feedback can be provided to respondents. Moreover, CD can be explored to find item characteristics not captured by the item difficulty parameters. Understanding from this exploration can be used to develop a better test or as a tool to detect potential latent clusters of items

Ignoring CD would result in averaging out item-wise variability across persons (equivalently, person-wise variability across items), and these unexplained variations would be incorrectly projected onto the measurement of intelligence (as a latent ability) or item characteristics. Therefore, considering and applying statistical models that can account for such CD is imperative for the development and application of precise intelligence tests, a prerequisite for intelligence research.

In this paper, we aim to provide an overview of the recently developed Latent Space Item Response Model (LSIRM; Jeon et al. 2021) as a model for CD and illustrate its advantages and utilities in the context of intelligence studies. The model measures latent abilities and item difficulties as main effects just as done in the Rasch model. However, it also quantifies and visualizes information derived from person-item interactions not explained by the Rasch model, enabling researchers to gain additional insights into persons and items in an intelligence test. Based on this information, the LSIRM further allows researchers to (1) investigate why lower response accuracy is observed for certain respondents than expected from the main effects, (2) capture the perceived difficulty of items varies across individuals, and (3) detect latent person/item clusters, or, group persons/items based on unexplained person-item interactions. The article will unfold as follows: In the next section, we provide an overview of the LSIRM model and its statistical inference method. Subsequently, we illustrate the advantages of LSIRM through two datasets related to intelligence. Finally, we conclude the paper with a discussion on CD in intelligence tests and the modeling thereof.

## 2. Latent Space Item Response Model

### 2.1. Model

In LSIRM, the Rasch model is combined with a latent space to capture unexplained interactions between persons and items through distance effects. Specifically, it is assumed that the general person ability $\theta_{p}\in R$ for person *p* (p=1,⋯,P) and the item difficulty $b_{i}\in R$ for item *i* (i=1,⋯,I) are placed on the same latent continuum, as in the Rasch model. They are supposed to capture the main person and the main item effects that underlie data. However, the Rasch model parameters are not able to capture interactions between persons and items. For instance, different persons may perceive the item difficulty for item *i* differently, even when they have the same ability level. As $b_{i}$ is constant, not a function of $\theta_{p}$, this effect cannot be appropriately accounted for.

A latent space is introduced to capture such interactions. In addition to $\theta_{p}$ and $b_{i}$, person *p* and item *i* have their positions, $\xi_{p}$ and $\zeta_{i}$, respectively, in a distinct *K*-dimensional latent space $R^{K}$. In this article, we use K=2 for easy exploration and visualization of an estimated latent space, as done in many previous studies (Handcock et al. 2007; Hoff et al. 2002; Jeon et al. 2021; Kang et al. 2023; Smith et al. 2019). Distances between persons and items defined on this latent space are supposed to account for residual dependencies unexplained by general characteristics such as $\theta_{p}$ and $b_{i}$. The model can be formally described with the following formula:

(1) $$logit\left(P\left(Y_{pi}=1|\theta_{p},b_{i},\xi_{p},\zeta_{i}\right)\right)=\theta_{p}+b_{i}+g\left(\xi_{p},\zeta_{i}\right).$$

In Equation (1), $g:R^{K}\times R^{K}\mapsto R$ is a real-valued function of the latent positions, representing the distance effect. With this function, distances between persons, between items, and between person and item can be calculated. In this article, we choose the following negative Euclidean distance with the tuning (weight) parameter γ as in the previous literature (Jeon et al. 2021; Kang et al. 2023).

(2) $$\begin{array}{cc} g(\xi_{p},\zeta_{i}) & =-\gamma \cdot d(\xi_{p},\zeta_{i})\\ d(\xi_{p},\zeta_{i}) & =\left|\right|\xi_{p},\zeta_{i}{\left|\right|}_{2}=\sqrt{\sum_{k=1}^{K}{\left(\xi_{pk}-\zeta_{ik}\right)}^{2}}, \end{array}$$

This implies that a lower response accuracy is predicted for a person who is located farther away from a specific item, compared to the accuracy value expected from person ability $\theta_{p}$ and item difficulty $b_{i}$.

The incorporation of the distance effect into the Rasch model also introduces an interesting integration of the two distinct response processes; cumulative and ideal point processes. If the item characteristic $b_{i}$ is redefined as the item difficulty parameter by taking its negative value (i.e., using $\theta_{p}-b_{i}$ instead of $\theta_{p}+b_{i}$ in Equation (1)), the Rasch model can be understood as a dominance model assuming the cumulative process. This process assumes that a response is more accurate and/or stronger if a person has a higher position than an item on their common latent continuum (i.e., if $\theta_{p}-b_{i}>>0$). This assumption is shared by many traditional IRT models. In contrast, unfolding IRT models (Roberts and Laughlin 1996; Roberts et al. 2000) for disagree-agree response scales (e.g., Likert scale) are based on the ideal point process as in multidimensional scaling (Borg and Gorenen 2005), assuming that a stronger agreement is predicted when person and item are closer in the latent continuum (i.e., when $\theta_{p}-b_{i}\approx 0$). The distance effect has a similar interpretation with the ideal point process in that the zero distance between person and item means there is no CD for this person-item pair. In other words, the ‘ideal’ point in a latent space corresponds to the ideal case in which main model parameters ($\theta_{p}$ and $b_{i}$) can perfectly explain the association between person *p* and item *i*, leaving no residual. This also gives a sensible interpretation of the negative distance effect on the response accuracy employed in Equation (1); the smaller the distance is, the closer the main model explanation comes to the ideal one, and the larger the distance is, the more residual interactions occur between persons and items.

It is also worth noting that the latent space is not an ability space. Observed responses have a fixed amount of (co)variations. The latent variable $\theta_{p}$ in the LSIRM accounts for some of them just as in the standard Rasch model, but there are almost always some residual (co)variations left (i.e., CD) in practice. Whereas the Rasch model leaves these residuals unexplained, the LSIRM further introduces the latent space to recognize interactive patterns of persons and items from this CD. In this sense, the ability space and the latent space are conceptually distinct. However, it does not mean that the latent variable and the latent positions are perfectly orthogonal and independent because there would be less (more) residual information to estimate latent positions as the latent variable explains a larger (smaller) proportion of associations between item responses.

Introducing additional latent variables (as in multidimensional IRT models) can reduce residuals, provided that CD is substantial and these additional factors can be theoretically validated. However, if there is no theoretical basis for additional factors, a latent space can be used as a tool to explore CD. Conversely, employing a unidimensional model in the presence of an unknown multidimensional latent structure in item responses may result in a large loss of data information. In this case, it can be expected that unspecified latent variables produce unexplained correlations between item responses and they can emerge as clusters in the latent space. This feature of the LSIRM will be illustrated in one of our empirical data analyses.

The introduction of the distance effect in the LSIRM yields some further theoretical and statistical distinctions from the traditional Rasch model, e.g., better ability estimation under omitted responses (Ho and Jeon 2023). Although we focus on what can be inferred from a visualized interaction map (i.e., a latent space) in this article, interested readers can refer to, e.g., Ho and Jeon (2023) and Jeon et al. (2021).

### 2.2. Inference

The model parameters can be estimated with Bayesian methods. In this article, we utilize **Stan** (Stan Development Team 2024) to fit the model with the Hamiltonian Monte Carlo (HMC). A **Stan** program to fit the LSIRM is provided in Section S1 in our Supplementary online Materials. Users can also find some alternative methods from the previous literature (Ho and Jeon 2023; Jeon et al. 2021; Luo et al. 2023). For Bayesian inference, we recommend the following prior specifications.

(3) $$\begin{array}{cc} \theta_{p} & ∼N(0,\sigma^{2}),\;\;p=1,\cdots ,P,\\ b_{i} & ∼N(0,5^{2}),\;\;i=1,\cdots ,I,\\ \sigma^{2} & ∼Half\text{-}Cauchy(25),\\ \xi_{p} & ∼MVN_{K}\left(0,I_{K}\right),\;\;p=1,\cdots ,P,\\ \zeta_{i} & ∼MVN_{K}\left(0,I_{K}\right),\;\;i=1,\cdots ,I,\\ \log(\lambda ) & ∼\left(1-\delta \right)\;N_{spike}\left(\mu_{\lambda 0},\sigma_{\lambda 0}\right)+\delta \;N_{slab}\left(\mu_{\lambda 1},\sigma_{\lambda 1}\right) \end{array}$$

where $N(\mu ,\sigma^{2})$ is a normal distribution with mean μ and standard deviation (SD) σ, Half-Cauchy(s) is a half-Cauchy distribution with scale *s*, $MVN_{K}\left(\mu ,\Sigma \right)$ is a *K*-dimensional multivariate normal distribution with mean vector μ and covariance matrix Σ. For identifiability constraints, means and SDs of latent positions $\xi_{p}$ and $\zeta_{i}$ are given 0 and 1, respectively.

An important feature in our prior choice is the slab-and-spike prior (Ishwaran and Rao 2005; Mitchell and Beauchamp 1988) given to the distance parameter λ. With this prior, the model can detect if there is substantial CD underlying data. When CD is large, the model is designed to choose the slab part, which has a little prior information so that λ can be estimated with a little bias. In contrast, when CD is small and can be ignored, the model chooses the spike part which is extremely dense at λ≈0 so that the distance effect and the latent space can be effectively removed from the model. With this model-selection feature, users can avoid greedily digging into residuals even when they do not provide meaningful and generalizable information. As in the previous latent space modeling (Jeon et al. 2021; Kang et al. 2023), we use $\mu_{\lambda 0}=-5$, $\mu_{\lambda 1}=0.5$, and $\sigma_{\lambda 0}=\sigma_{\lambda 1}=1$. Then, when the slab is chosen (δ=1), the prior distribution of λ|δ=1 has a mean of 2.718, mode of 0.607, and SD of 3.565, allowing the estimation of λ without noticeable bias. If the Rasch model is sufficient for data, the spike prior is chosen, which has a mean of 0.011, mode of 0.002, and SD of 0.015, shrinking λ to zero.

The distance effect has translational, reflective, and rotational invariances with respect to latent person and item positions, which incurs an identifiability issue for position parameters (Jeon et al. 2021; Kang et al. 2023). This can be resolved with Procrustes matching (Borg and Gorenen 2005; Friel et al. 2016; Gower 1975), which is a standard method in multidimensional scaling to match two configurations. Specifically, the posterior sample of latent positions with the highest posterior density can be chosen as a reference set. Then, Procrustes matching can be applied to the other posterior samples of latent positions to match the reference set as much as possible, while maintaining the distances between persons, between items, and between persons and items. After this procedure, point estimates of the latent positions can be obtained.

For the following empirical applications to illustrate the utilities of the LSIRM in intelligence studies, we fitted the model to data with the described Bayesian estimation method. We ran 3 Bayesian chains with 1500 iterations for each, but discarded the first 500 for burn-in. We assessed the convergence of chains with visual inspection of posterior distributions (trace plots and posterior densities) as well as the potential scale reduction factor ($\hat{R}$; Gelman 1996; Gelman et al. 2013) with the cutoff value of 1.1. All $\hat{R}$ values were smaller than 1.01 and we did not find any convergence issue. Relevant figures are provided in Section S2 in our Supplementary online Materials.

## 3. Empirical Illustrations

### 3.1. Data Description

In order to demonstrate the advantages of the LSIRM in the context of intelligence measurement, we analyzed two datasets. The first dataset was from a Vocabulary-based Intelligence Quotient Test (VIQT), obtained from an online data repository called Open Source Psychometrics Project (https://openpsychometrics.org/, can be accessed on 27 March 2024). There were 45 items in VIQT. Each test item presented five vocabularies and asked a respondent to choose two of them with the same meaning. The raw dataset has 12173 respondents but we randomly selected P=1000 respondents for our demonstrative data analysis.

The second dataset was based on the Inductive Reasoning Developmental Test (IRDT) used in Golino and Epskamp (2017) to demonstrate the strength of an exploratory graph analysis (EGA) method in detecting latent dimensions of test items. The test aimed to measure seven hierarchical and sequential developmental stages of inductive reasoning, each measured by 8 items (I=56 items in total). Labels for the seven stages are as follows:

- Items 1–8: Pre-Operational
- Items 9–16: Primary
- Items 17–24: Concrete
- Items 25–32: Abstract
- Items 33–40: Formal
- Items 41–48: Systematic
- Items 49–56: Metasystematic

There were P=1803 respondents in the dataset we obtained from the link provided in Golino and Epskamp (2017, see p. 15). In the previous study, it was described that both a 7-factor confirmatory factor analysis (CFA) model and a bi-factor model with one global factor and 7 specific factors can provide adequate fits to the data. Also, the dataset was used to demonstrate that EGA can detect the same 7-dimensional latent structures of the test items as designed in the test development.

The second dataset is not appropriate for the LSIRM (neither for the standard Rasch model) because the model assumes a unidimensional latent ability represented by $\theta_{p}$ whereas the data is known to have seven underlying factors. We intentionally chose this dataset for the same illustrative purpose as in Golino and Epskamp (2017). When a test has a multidimensional latent structure but it is not informed a priori, a typical unidimensional model (e.g., Rasch model) could likely be employed to fit the data. In this case, necessary but unspecified factors produce residual dependence, yielding a bad model fit. If a latent space is added to the Rasch model (i.e., LSIRM), unexplained correlations due to unspecified factors may emerge as systematic patterns or clusters of estimated latent positions. This feature will be illustrated below.

### 3.2. Main Analysis

#### 3.2.1. Positions in the Estimated Latent Space

**Figure 1:**
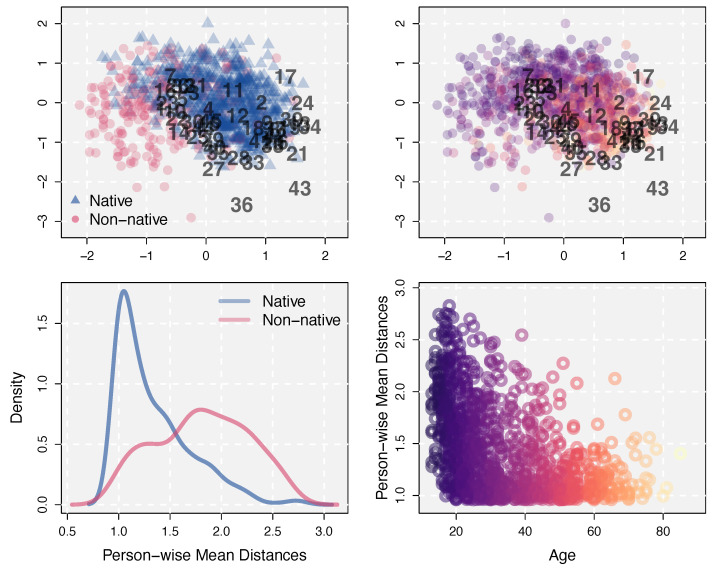
**Top**: The estimated latent space for the VIQT data. Persons are color-coded according to whether their first language is English or not (**left**) or according to their age (**right**). **Bottom**: Person-wise mean distances to items (averaged across items). Densities for English native speakers and non-natives (**left**) and a scatter plot of distances as a function of age (**right**). For age, yellower-brighter represents older whereas purpler-darker represents younger.

For the VIQT dataset, the slab-and-spike prior of the LSIRM detected substantial unexplained interactions between persons and items with P(δ=1|Y)=0.93 and $\hat{\lambda }=1.54$. To explore these interactions, we generated the interaction map with the estimated latent positions ${\hat{\xi }}_{p}$’s and ${\hat{\zeta }}_{i}$’s, shown in the top panels in Figure 1. In the interaction map, dots represent persons whereas numbers indicate items. To demonstrate that latent person positions can provide further information on persons in test data, we associated some external variables in the dataset with the estimated latent person positions. For an illustrative purpose, we color-coded persons in the latent space by those external variables. In the top-left panel, we used information regarding whether English is the mother tongue of a person (blue triangle) or not (red circle). The latent space shows that English native speakers are generally located on the right side of the space, whereas non-native speakers are on the left side, having larger distances on average to the VIQT items. Although there are some overlaps, the two groups have a clear separation with respect to average distances to items, as shown by distance densities in the bottom-left panel. Judging from person-wise mean accuracy, persons whose first language is not English performed generally worse (M=0.79, SD=0.15) than English native speakers (M=0.57, SD=0.18) in the VIQT conducted in English. However, their internal language-related ability (i.e., $\theta_{p}$) is not the sole reason for this difference in performance; the language choice of the test also influences the test performance, which cannot be captured by the traditional Rasch model unless this source is specified a priori and the Rasch model is modified to capture it.

The top-right panel in Figure 1 shows the same latent space, but persons are color-coded according to their ages. The age range in the data was [13,85]. In the figure, yellower-brighter represents older whereas purpler-darker represents younger. The same color code was used in the bottom-right panel in which age is plotted on the x-axis against person-wise mean distances to items (averaged across items) plotted on the y-axis. The figure shows that most older people have shorter distances to most items whereas younger people generally are located farther away from items and also have larger individual differences. This result implies that vocabularies in some items are too difficult for younger people, more than for older people. For instance, items 36 and 43, which have the largest average distances to persons, have the following vocabularies as response options (the correct answers are bolded).

- Item 36: fulminant doohickey ligature **epistle letter**
- Item 43: fugacious vapid **fractious querulous** extemporaneous

Presumably, the trend of younger people having lower accuracy for these items was because they have fewer chances in their lives to get exposed to and learn those vocabularies. The estimated latent space can account for this by the negative distance effect on the response accuracy. It is noteworthy that item 43 was the most difficult item but item 36 was the 10-th out of I=45, in terms of item-wise accuracy. Thus, the estimated distance effects are separable from the general item difficulties. The interaction map of the VIQT dataset, shown in two ways with Figure 1, illustrates potential sources of CD and how a latent space can capture these unexplained interactions between persons and items.

#### 3.2.2. Varying Item Difficulties across Persons

It was described in the previous section that some vocabulary items can be more difficult for younger people compared to older people who have longer individual histories of learning words. In fact, the same test items can be more or less difficult for different respondents, which is one of the primary sources of CD (Jeon et al. 2021; Kang et al. 2023). The Rasch model, which assumes CI between responses, is not able to account for this potential between-person variability in item difficulty. The LSIRM, in contrast, is able to quantify and visualize this variation with estimated distance effects. After controlling for the person latent ability $\theta_{p}$, the LSIRM determines response accuracy as a function of $b_{i}-\gamma \cdot d\left(\xi_{p},\zeta_{i}\right)$. For item *i*, this represents item effect plus variations across persons as different persons have different distances to the same item. These quantities can be compared with the item difficulty estimates of the Rasch model.

**Figure 2:**
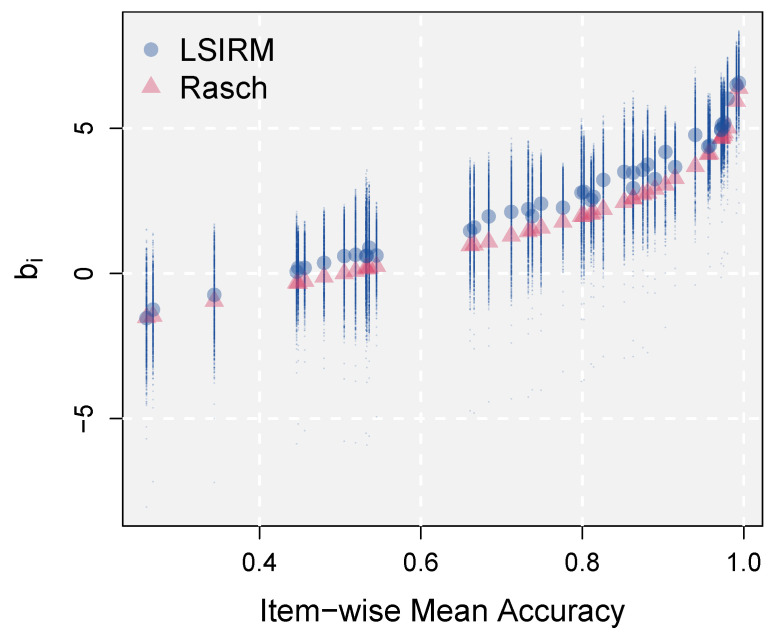
Across-person variations in perceived item difficulty.

In Figure 2, the Rasch estimates of item difficulty (red triangles) are plotted on the y-axis against observed item-wise mean accuracy values on the x-axis. The item effects estimated by the LSIRM are plotted in two ways. First, the distance effects were averaged over respondents, but by item separately, and added to ${\hat{b}}_{i}$. That is, ${\hat{b}}_{i}-\hat{\gamma }\cdot \frac{1}{P}\sum_{p=1}^{P}d\left({\hat{\xi }}_{p},{\hat{\zeta }}_{i}\right)$ was obtained and plotted against observed item-wise mean accuracy (blue dots). In addition, the raw distance effects ${\hat{b}}_{i}-\hat{\gamma }\cdot d\left({\hat{\xi }}_{p},{\hat{\zeta }}_{i}\right)$ were plotted, which generated a vertical line of dots for each item. This can be considered a full interval of variability in item difficulty across persons, estimated based on the distance effects implemented in the LSIRM.

The figure shows that the estimated item effects by the Rasch model and the LSIRM were generally consistent. However, as shown by the LSIRM results, the item effects exhibited large variability across persons. These individual differences in how persons perceive item difficulty can be used to provide personalized feedback and study item characteristics (see more from Section 3.2.4). Also as shown in the previous section, this distance-based information can be further studied in the latent space with external variables to derive information for diagnosis and evaluation. This analysis was based on the slab-and-spike prior of the LSIRM, which chose the slab part, supporting that there were substantial interactions between respondents and items and these were captured by the latent space. Therefore, across-person variations in item difficulty shown in Figure 2 were statistically supported. The Rasch model cannot capture these variations, leaving valuable information on respondents and items unanalyzed without a thorough examination.

#### 3.2.3. Studying Additional Item Information and Latent Structure: Unspecified Factors as a Data Source of Conditional Dependence

The IRDT dataset was analyzed by Golino and Epskamp (2017) to demonstrate how to determine the number of latent dimensions of test items based on EGA. The method used partial correlations between responses to detect clusters. The result revealed 7 dimensions, each measured by 8 items (see Figure 8 in Golino and Epskamp 2017).

Assuming that the underlying latent structure of the test is unknown, the Rasch model may be employed to estimate the latent abilities of respondents. However, it is likely that the Rasch model cannot provide an adequate fit to the data due to unspecified factors, which produce substantial CD. The purpose of analyzing the IRDT dataset with the LSIRM is to illustrate that this source of CD (misspecification of a latent structure) can be detected by a structure that latent positions formulate in an estimated latent space.

**Figure 3:**
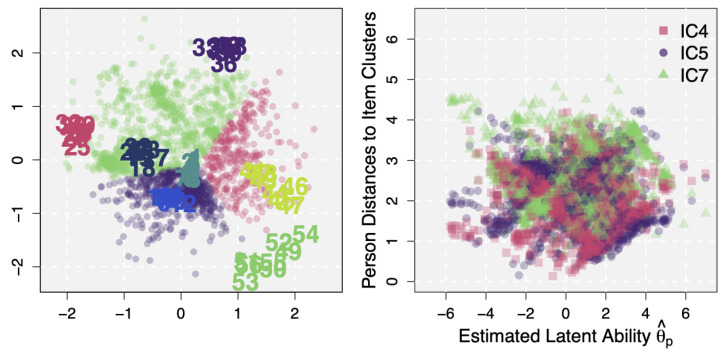
**Left**: The estimated latent space for the IRDT data. Persons are color-coded according to the item clusters for which they have the largest distance. **Right**: A scatter plot of the estimated latent abilities on the x-axis against the estimated person-wise mean distances to selected item clusters (red: 4, purple: 5, and green: 7).

The left panel of Figure 3 shows the latent space of the IRDT dataset, estimated by the LSIRM. The model identified significant CD, with P(δ=1|Y)=0.91 and $\hat{\lambda }=2.07$. This CD is represented by distances on the latent space between persons, between items, and/or between persons and items. First of all, numbers in the latent space represent the estimated positions of test items (circles represent persons, but they will be studied in Section 3.2.4). They are color-coded according to the latent dimension assignments obtained by the previous EGA approach. Although the first three clusters (items 1–8, 9–16, and 17–24) were located relatively close to each other, items in the latent space generally show the same item clusters as found by EGA.

**Figure 4:**
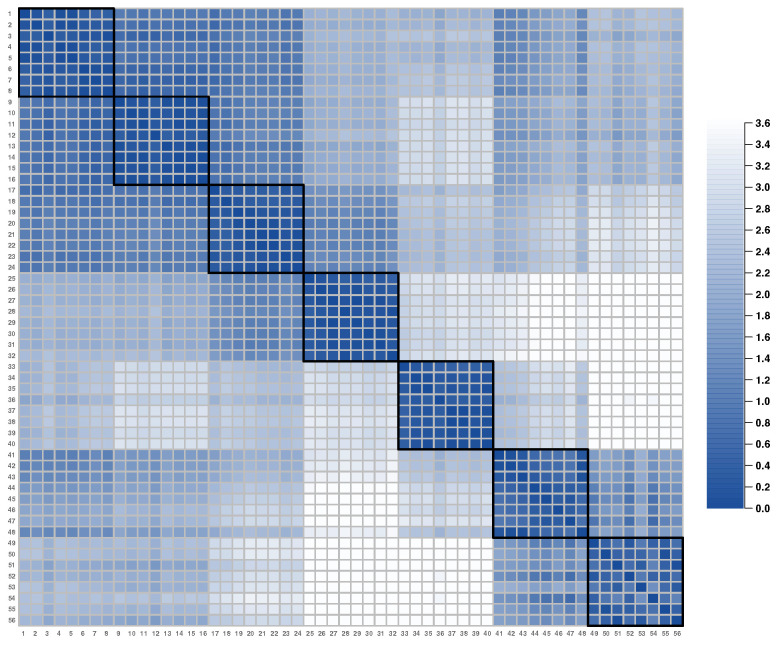
Inter-item distances estimated by the LSIRM.

Additional support for the latent item clusters can be obtained by computing inter-item distances $d(\zeta_{i},\zeta_{j})$ (for all *i* and *j*, i≠j) on the latent spaces. This calculation produces an (I×I) item distance matrix, which is visualized in Figure 4. In the figure, the distance values are color-coded according to the legend on the right side. Although there are some clusters closely located with each other, the figure shows a block-diagonal structure, which is consistent with the latent dimensions obtained in Golino and Epskamp (2017).

Beyond the simple clustering of items, we can further study similarities and dissimilarities between clusters based on their distances. To this end, we first defined a cluster center simply as a mean latent position ${\bar{\zeta }}_{.kc}^{*}$ (k=1,2 and c=1,⋯,7) of items within a cluster, with which inter-cluster distances can be calculated. Table 1 presents the calculated cluster positions and distances from the IRDT dataset. Although the relationship between item clusters can be visualized as done in the left panel of Figure 3, these calculated values can further quantify whatever is seen for item clusters in the latent space. Also, as this observation is from the latent space, the findings must be attributed to residual dependence, not the main person and item effects. The figure and the calculated values imply that item clusters 1–3 are generally similar to each other in terms of CD whereas the other clusters, particularly 4, 7, and 8, have their residual idiosyncrasies (e.g., see ‘Mean’ item distances in Table 1). This way of study is not feasible in EGA and the bifactor model (which were shown to well describe the systematic latent structure under the IRDT dataset in Golino and Epskamp 2017) because (1) EGA is able to estimate edges between nodes (items) but not potential node clusters and (2) estimated (specific) factors in the bifactor model are usually assumed to be uncorrelated, and thus it is hard to formally and quantitatively investigate the relationship node clusters and/or specific factors. A multidimensional latent variable model, which is another suitable approach for the IRDT dataset, can use factor correlations to quantitatively study associations between item clusters. An advantage of the LSIRM over this approach is that item clusters and their relationships can be visually inspected (as in the left panel of Figure 3) and unexplained person-item interactions can be further explored (which will be described in the next section).

#### 3.2.4. Person-Item Interactions from Conditional Dependence and Generation of Personalized Feedback

A further advantage of the LSIRM is that the latent space provides information regarding respondents as well as items. In the estimated latent space of the IRDT dataset (the left panel of Figure 3), circles represent P=1803 persons. Given these latent person positions, distances between a specific person and items can be computed, resulting in I=56 distance values for a single person. Then, these distances can be averaged across items but separately by item clusters, resulting in 7 cluster distances for each person. In this way, we can quantify person-wise amounts of CD for different item clusters and identify item clusters for which a person has the largest distance. That is, item clusters with the largest CD for each person can be found for prospective analysis of person/item characteristics and derivation of diagnostic information.

This analysis was conducted and the estimated person positions in the latent space were color-coded according to the item clusters with the largest distances (i.e., using the color of the item cluster farthest to each person). Roughly, persons were categorized into three groups; those with the largest distances to the item clusters 4 (red), 5 (purple), and 7 (green). A larger person-cluster distance means that, for items in the corresponding cluster, the person did not perform as well as what was expected from estimated ability and item difficulties (i.e., the negative distance effect of CD). This information can be used to detect item types that a person specifically finds difficult to solve. For example, item cluster 4 (Abstract; red-colored items 25–32) in the latent space had a mean accuracy of 0.484, which was at the intermediate level compared to the other item clusters. In fact, item clusters 5–7 (items 33–56) were generally more difficult than item cluster 4 (with mean accuracy values of 0.268, 0.098, and 0.035, for item clusters 5, 6, and 7, respectively). However, 324 of 1803 respondents were color-coded red in the figure, meaning that they performed particularly worse for item cluster 4. That is, these red-coded respondents showed poorer accuracies for items in cluster 4 although they performed relatively well for ‘even more difficult’ items. This can be potentially attributed to their own weakness to item-specific natures of items in cluster 4 and/or an aspect of intelligence measured by these items.

It should be noted that this was not because they have low latent abilities. The mean of the estimated latent ability ${\hat{\theta }}_{p}$ was 0.406 (SD=1.922) for the respondents farthest from item cluster 4 and 0.452 (SD=1.982) for the other respondents, showing no significant difference. Accordingly, the respondents farthest from item cluster 4 had a similar overall mean accuracy (0.494) for all items as the other respondents (0.525), but their mean accuracy for item cluster 4 was much lower (0.139, compared to the mean accuracy of 0.497 for the others). Also, in the right panel of Figure 3, ${\hat{\theta }}_{p}$ is plotted on the x-axis against person-wise mean distances to some selected item clusters, 4 (red squares), 5 (purple dots), and 7 (green triangles). The scatter plot shows no noticeable pattern, meaning that the distance effects are not associated with the latent abilities and so they account for variations in data unexplained by the main person effects.

The same analysis can be carried out at the individual level (rather than groups of respondents). For instance, in Table 2, four representative persons with similar latent abilities are selected and their ID, estimated latent abilities, latent positions, distances to item clusters, and overall (person-wise) response accuracy are presented. As intended, these persons had similar ability levels as shown in the second column of the table. However, their latent positions and distances to item clusters were largely different. Roughly speaking, persons 64 and 1359 were located at the top-left side of the latent space whereas persons 1653 and 1655 were at the bottom-right side (judging from their ${\hat{\xi }}_{p1}$ and ${\hat{\xi }}_{p2}$). Accordingly, for persons 64 and 1359, their farthest item cluster was cluster 7, whereas it was clusters 5 and 4 for persons 238 and 957, respectively. This example demonstrates that even persons with a similar level of underlying latent abilities can have varying patterns of CD due to their strengths and weaknesses to different types of test items.

Along with general feedback based on the estimated latent ability, which is also available in traditional CI models (e.g., the Rasch model and 2PLM), a CD model such as the LSIRM can further provide personalized feedback using this item-specific information (i.e., distance to a specific item or item cluster). For example, for persons 64 and 1359, it can be deduced that they performed well for items in the first four clusters but not for items in clusters 5–7. This information can be used to investigate for which aspect of the intelligence a respondent has a weakness. Similarly, CD also can be informative for test designers. For instance, if there are some items that a specific group of respondents (e.g., those with different cultural backgrounds, different first languages, etc.), a test may need to be redesigned by excluding or modifying such items.

Note that the above illustration of generating personalized feedback based on person-cluster distances was based on the assumption that the underlying latent structure of the IRDT dataset was unknown. Even when the optimal number of factors and the factor structure are known, however, the same strategy can be applied. With multidimensional factors, a latent space and person/item positions can be estimated. If (co)variations of data cannot be sufficiently explained by multiple factors and item parameters, residual associations can be captured by the distance effects. The distances can be further studied to detect potential item clusters, person clusters, and person-item interactions, which provide personalized feedback and diagnosis for respondents. It should be further noted that this way of finding feedback can be conducted with individual items, even when no noticeable item clusters are detected in an estimated latent space.

## 4. Discussion

### 4.1. Summary

In this article, we introduced the LSIRM, a new IRT model integrated with a network analysis approach. The LSIRM assumes that persons and items are located in a metric space called a latent space. Latent positions are obtained separately and independently from latent abilities and item parameters. In contrast to the traditional Rasch model, the LSIRM can account for not only the main person and item effects (i.e., general abilities and item difficulties) but also their interactions by means of distances between persons and items on the estimated latent space. It is assumed that, if a person has a large distance from an item, the person performs worse for the item compared to the accuracy predicted by the main effect parameters in the Rasch model.

The Rasch model assumes CI, meaning that correlations between responses can be fully captured by latent abilities and item parameters and there is no more dependence between responses after the main person and item effects are controlled for. The slab-and-spike prior imposed on the tuning parameter in the LSIRM can examine if this assumption is reasonable. If there is a little CD underlying response data, the spike part shrinks the distance effect to zero, reducing the model to the typical Rasch model. In contrast, the slab part estimates the distance effect with the minimum bias if data imply substantial CD. Then, the CD can be visualized and inspected with the interaction map. This CD represents unexplained interactions between persons and items. For example, the same item can be more or less challenging for different respondents regardless of their ability levels. Some respondents may have difficulties understanding and solving a specific type of test item. All these interactions can affect response accuracy, which cannot be fully captured by the latent ability $\theta_{p}$ and the item difficulty $b_{i}$ in the Rasch model.

### 4.2. Advantages of the LSIRM

Unexplained interactions between persons and items can be associated with external variables to study the nature and sources of CD. In our analysis of the VIQT dataset, the estimated latent person positions on the latent spaces were linked with (1) whether English is the first language of persons and (2) age. It was found that Non-native English speakers and younger people generally have larger distances to vocabulary test items carried out in English. The lower response accuracy of these respondents, beyond the prediction by the main person and item effects in the Rasch model, can be (at least partially) attributed to CD. Then, this CD can be further studied with external variables to obtain a better understanding of variations in respondents’ performance in an intelligence test. In this sense, person positions on the latent space and their associations with external variables (along with the estimated latent abilities) can be used to provide person-customized diagnosis and feedback.

Another view on CD was examined with item-wise variability in difficulty across respondents. Most of the traditional IRT models and their applications assumed that item difficulty is a global property, meaning that it is constant for all respondents. However, significant CD implies that this is not the case. The LSIRM model can account for this variability as item-wise distances to *P* respondents. These distances combined with the item effect estimates can provide an interval of item difficulty across respondents (as done in Figure 2. The averaged item effect estimates over respondents have a great consistency with the estimates from the Rasch model. Additional item effect intervals (not provided by the Rasch model) can provide quantifications of individual differences in perceived item difficulties.

An estimated latent space can also be explored to detect the underlying latent dimensions of items. Potential item clusters can be found with the visualization of an interaction map and computed distances between items. This is not more than what can be already achieved by the existing methods such as traditional exploratory factor analysis and recent advancements in graph analysis (e.g., EGA as used in Golino and Epskamp 2017). However, there are several important differences. The first difference is in how to inspect similarities and dissimilarities of detected item clusters. An estimated latent space facilitates, with visualization of latent positions and quantification of CD, a study of common characteristics and idiosyncrasies of item clusters, which is not or only partially available in the previous methods. This can be helpful in better understanding how items work in the measurement procedure and refining test items for more accurate and precise measurements.

The second difference is the primary source investigated to find the optimal latent dimensions. For example, Factor analysis and multidimensional IRT models utilize full correlational structures underlying item responses to determine the best number of factors. EGA relies on the partial correlations of responses to generate graphical representations of item networks, i.e., the resulting latent dimension is based on bivariate associations of item responses after controlling for information provided by all the other items. In contrast, the LSIRM uses residual associations of item responses after controlling for the latent ability (similar to residual covariance in a single-factor confirmatory factor analysis). In this sense, the LSIRM is aligned with a bi-factor model in that the latent ability works as a general factor and a latent space captures influences of potential specific factors. It is worth noting that a latent space can be incorporated into a multidimensional IRT model, which constructs a potential extension of the current LSIRM. In this case, the revised model can be a more appropriate candidate for the tests known to measure multiple factors. Unlike the previous multidimensional models, however, this model can explore unexplained interactions between persons and items and detect unspecified factors if any. This application, of course, should be performed with adequate management against overfitting, such as the slab-and-spike prior or other regularization techniques. This kind of multidimensional extension is currently under development.

Last but not least, unlike the previous approaches to finding optimal latent dimensions, the LSIRM can further provide information on persons and their associations with items, e.g., for which items a person specifically performs better or worse. The general ability measure, which is independent of item characteristics, cannot capture these potential variations. In other words, even persons with similar levels of latent abilities can have different patterns of interactions with test items which are manifested as their differences in item response profiles. Their distances to items can be utilized to study these unexplained associations between persons and items. Also, as item clusters, potential person groups can be defined (or person clusters can be identified) based on person-to-item distances or person-to-person distances. Figure 3 was an example of this approach in which person groups were defined based on item groups that persons have the smallest or the largest distances on the latent space.

### 4.3. Related Modeling Approaches

The interaction map generated by the LSIRM quantifies and visualizes CD underlying item responses. By definition, CD is residual variations, after controlling for person and item effects with person-wise and item-wise model parameters. This means that residual variations can be reduced by introducing more parameters in a model. For example, the 2PLM can capture systematic variations in the data more than the Rasch model as previously described in our introduction, based on the product term of item discrimination parameters and latent abilities. However, this does not necessarily mean that all valuable information underlying data has been extracted and analyzed. Whether the implementation of the item discrimination parameters suffice to account for variations between item responses or there still is substantial CD underlying data can be studied by incorporating a latent space into the 2PLM (e.g., Go et al. 2022).

Traditionally in the IRT literature, CD has typically been called the differential item functioning (DIF; Magis et al. 2010) and various methods to detect DIF have been proposed and examined. For example, the IRT-based likelihood ratio (IRT-LR) tests (Thissen et al. 1993) attempt to detect if there is a significant difference in item characteristics between groups by comparing an IRT model and a restricted model in which some item parameters are constrained to be equal across the groups. Likewise, most of the existing DIF tests focus on group differences in item properties (c.f., Molenaar 2021). An advantage of studying CD based on the LSIRM is that this approach is free from such a restriction; with a visualized latent space and calculated person-item distances, CD can be further studied with group variables, continuous variables, or even without an external reference variable. In Figure 1, we illustrated an exploratory study of CD with (1) whether the first language is English or not (group variable) and (2) age (can be regarded as continuous). A study of person and item interactions can also be performed without such variables, e.g., using person-item distances. For example, if a researcher is interested in potential across-person differences in responses to item *i*, the distance to this item can be computed for every person and compared. If wanted, a group is defined based on this distance (as done in Figure 3, with item clusters as targets) for further analyses such as generating feedback for persons who performed particularly worse for that item. This analysis can be expanded to multiple items or item clusters, and can also incorporate extrinsic (categorical and/or continuous) variables of interest. It could be a concern that this approach might be greedily applied to find arbitrary ‘bogus’ groups. However, the model can estimate meaningfully distributed latent positions only when CD is substantial, thanks to the slab-and-spike prior. Also, the LSIRM was not invented solely to test a potential group difference in item characteristics and was not meant to be an alternative to the traditional DIF testing methods. Instead, it is a more liberal approach to generally explore conditional dependence, which could be due to (but not restricted to) differences in item characteristics between pre-specified groups.

In recent times, interest in CD has extended to joint modeling of responses and response times (RTs) At the initial stage of such joint modeling approaches, it was prevalent to assume CI between responses and RTs (as well as CI between responses and CI between RTs), after the hierarchical framework by van der Linden (2007). However, recent evidence suggests that many test data do not support such assumptions, leading to an increase in studies modeling observed CD between responses and RTs (e.g., Bolsinova et al. 2017c, 2017a; Bolsinova and Molenaar 2018; Goldhammer et al. 2014, 2015; Meng et al. 2015; Partchev and De Boeck 2012; van der Linden and Glas 2010; van Rijn and Ali 2017; Wang and Xu 2015). Some prior research has outlined the theoretical mechanisms behind the emergence of CD between responses and RTs, such as speed-accuracy trade-offs and fast/slow guessing (e.g., Bolsinova et al. 2017b; De Boeck et al. 2017). Furthermore, there have been studies that combine mathematical modeling and psychological measurement models to examine some of these theoretical mechanisms based on formal models and data analysis (e.g., Kang et al. 2022a, 2022b). The recently proposed Latent Space Diffusion Item Response Theory Model (Kang et al. 2023) employs a similar approach to LSIRM; It analyzes variations in responses and RTs from psychological/educational tests based on cognitive processes and cognitive components involved in decision-making/problem-solving processes, and simultaneously, attempts to capture and visualize CD between item responses, between RTs, and between responses and RTs through latent space and distance effects.

Both the standard Rasch model and the LSIRM, applied to intelligence-relevant datasets, assume that a latent variable represents intelligence as traditionally done in psychometric modeling. Alternative explanations for this include mutual connections or a network of items as a representation of intelligence and it has been shown that they can also produce observed associations of variables as a latent variable representation (Epskamp et al. 2018; Marsman et al. 2018; van der Maas et al. 2006). A version of the LSIRM more consistent with the network approach can be developed and examined. For example, the latent variable $\theta_{p}$ in Equation (1) can be excluded, producing a new logit probability formula $logit\left(P\left(X_{pi}=1|...\right)\right)=b_{i}-\gamma \cdot d\left(\xi_{p},\zeta_{i}\right)$. This might be viewed as having some consistency with the Ising model representation of item responses in network psychometrics in that $b_{i}$ works as the threshold parameter (regarding whether item *i* prefers which of the binary values it can take) and the distance term plays the role of the network parameter (pairwise interaction between two item responses, regarding whether they take the same value or not). In this case, latent positions of persons and items should be estimated based on (co)variations of item responses after controlling for item effects only. A latent trait may emerge as densely distributed item positions, as presumed in the network approaches. One big difference is that, unlike the network psychometrics, this latent space approach can study not only a network of items, but also a network of persons as well as a person-item network. A formal examination of this possibility and a study of the potential consistency mentioned above is not within the scope of the current article. However, this can be an interesting topic to investigate in a follow-up development of the latent space approach.

### 4.4. Conclusion

The use of latent space allows for a more comprehensive analysis of respondents and items, providing in-depth information for evaluation and diagnosis. As exemplified above with joint modeling of responses and RTs, integration of latent space with traditional psychometric models is applicable beyond the confines of item responses in traditional intelligence testing, offering new avenues for measuring and studying intelligence in diverse contexts. We anticipate that such an integrative approach will serve as a catalyst for advancements in intelligence research across various applications.

## Figures and Tables

**Table 1:** Item clusters defined based on the estimated item positions on the latent space and their inter-cluster distances.

IC	Item Positions		Item Distances							
	${\bar{\zeta }}_{.1c}^{*}$	${\bar{\zeta }}_{.2c}^{*}$	1	2	3	4	5	6	7	Mean
1	0.168	−0.174	-	0.673	0.902	2.116	2.316	1.476	2.178	1.601
2	−0.207	−0.733		-	0.977	2.054	2.960	1.840	2.094	1.766
3	−0.685	0.120			-	1.214	2.422	2.373	2.988	1.813
4	−1.828	0.529				-	3.008	3.584	4.143	2.686
5	0.758	2.065					-	2.677	3.989	2.895
6	1.613	−0.471						-	1.370	2.220
7	1.570	−1.840							-	2.794

IC: Item Cluster, Mean: Mean distances to other item clusters (computed for each cluster).

**Table 2:** Estimated latent abilities, latent positions, person-wise mean distances to item clusters, and person-wise mean response accuracy for some selected persons.

Person	Ability	Positions		Person-Wise Mean Distances to Item Clusters							Acc
	${\hat{\theta }}_{p}$	${\hat{\xi }}_{p1}$	${\hat{\xi }}_{p2}$	1	2	3	4	5	6	7	
64	1.043	−1.227	0.656	1.622	1.723	0.762	0.615	2.434	3.055	3.749	0.571
1359	1.155	−1.285	1.016	1.877	2.054	1.078	0.730	2.296	3.257	4.038	0.571
1653	1.227	1.015	−1.775	1.812	1.607	2.546	3.660	3.849	1.435	0.559	0.411
1655	1.060	1.424	−0.976	1.491	1.650	2.377	3.584	3.113	0.539	0.877	0.446

Acc: Person-wise mean response accuracy.

## Data Availability

The original VIQT data can be downloaded from https://openpsychometrics.org/_rawdata/ (accessed on 27 March 2024). A link to download the IRDT data can be found in p. 15 in Golino and Epskamp (2017).

## Supplementary Materials

The following supporting information can be downloaded at: https://www.mdpi.com/article/10.3390/jintelligence12040038/s1, Figure S1: Histogram of Potential Scale Reduction Statistics ($\hat{R}$), from the LSIRM fit to the VIQT dataset (left) and the IRDT dataset (right); Figure S2. Trace Plots of Parameters for Randomly Selected Persons and Items in the VIQT Data Analysis; Figure S3. Trace Plots of Parameters for Randomly Selected Persons and Items in the IRDT Data Analysis.

## Abbreviations

The following abbreviations are used in this manuscript:

CD	Conditional Dependence
CFA	Confirmatory Factor Analysis
CI	Conditional Independence
DIF	Differential Item Functioning
EGA	Exploratory Graph Analysis
HMC	Hamiltonian Monte Carlo
IRDT	Inductive Reasoning Developmental Test
IRT	Item Response Theory
LR	Likelihood Ratio
LSDIRT	Latent Space Diffusion Item Response Theory Model
LSIRM	Latent Space Item Response Model
RT	Response Time
SD	Standard Deviation
VIQT	Vocabulary-based Intelligence Quotient Test
2PLM	Two-Parameter Logistic IRT Model

## References

[B1-jintelligence-12-00038] Binet Alred, Simon Théodore, Dennis Wayne (1948). The development of the binet-simon scale, 1905–1908. Readings in the History of Psychology.

[B2-jintelligence-12-00038] Bolsinova Maria, Molenaar Dylan (2018). Modeling nonlinear conditional dependence between response time and accuracy. Frontiers in Psychology.

[B3-jintelligence-12-00038] Bolsinova Maria, Tijmstra Jesper, Molenaar Dylan (2017a). Response moderation models for conditional dependence between response time and response accuracy. British Journal of Mathematical and Statistical Psychology.

[B4-jintelligence-12-00038] Bolsinova Maria, Tijmstra Jesper, Molenaar Dylan, De Boeck Paul (2017b). Conditional dependence between response time and accuracy: An overview of its possible sources and directions for distinguishing between them. Frontiers in Psychology.

[B5-jintelligence-12-00038] Bolsinova Maria, De Boeck Paul, Tijmstra Jesper (2017c). Modelling conditional dependence between response and accuracy. Psychometrika.

[B6-jintelligence-12-00038] Borg Ingwer, Gorenen Patrick J. F. (2005). Modern Multidimensional Scaling: Theory and Applications.

[B7-jintelligence-12-00038] De Boeck Paul, Chen Haiqin, Davison Mark (2017). Spontaneous and imposed speed of cognitive test responses. British Journal of Mathematical and Statistical Psychology.

[B8-jintelligence-12-00038] Epskamp Sacha, Maris Gunter, Waldorp Lourens J., Borsboom Denny, Irwing Paul, Booth Tom, Hughes David J. (2018). Network psychometrics. The Wiley Handbook of Psychometric Testing: A Multidisciplinary Reference on Survey, Scale and Test Development.

[B9-jintelligence-12-00038] Friel Nial, Rastelli Riccardo, Wyse Jason, Raftery Adrian E. (2016). Interlocking directorates in irish companies using a latent space model for bipartite networks. Proceedings of the National Academy of Sciences.

[B10-jintelligence-12-00038] Gelman Andrew, Gilks Walter R., Richardson Sylvia, Spiegelhalter David J. (1996). Inference and monitoring convergence. Markov Chain Monte Carlo in Practice.

[B11-jintelligence-12-00038] Gelman Andrew, Carlin John B., Stern Hal S., Dunson David B., Vehtari Aki, Rubin Donald B. (2013). Bayesian Data Analysis.

[B12-jintelligence-12-00038] Go Dongyoung, Park Jina, Park Junyong, Jeon Minjeong, Jin Ick Hoon (2022). lsirm12pl: An r package for latent space item response modeling. arXiv.

[B14-jintelligence-12-00038] Goldhammer Frank, Naumann Johannes, Greiff Samuel (2015). More is not always better: The relation between item response and item response time in raven’s matrices. Journal of Intelligence.

[B13-jintelligence-12-00038] Goldhammer Frank, Naumann Johannes, Stelter Annette, Tóth Krisztina, Rölke Heiko, Klieme Eckhard (2014). The time on task effect in reading and problem solving is moderated by task difficulty and skill: Insights from a computer-based large-scale assessment. Journal of Educational Psychology.

[B15-jintelligence-12-00038] Golino Hudson F., Epskamp Sacha (2017). Exploratory graph analysis: A new approach for estimating the number of dimensions in psychological research. PLoS ONE.

[B16-jintelligence-12-00038] Gower John C. (1975). Generalized procrustes analysis. Psychometrika.

[B17-jintelligence-12-00038] Handcock Mark S., Raftery Adrian E., Tantrum Jeremy M. (2007). Model-based clustering for social networks. Journal of the Royal Statistical Society: Series A (Statistics in Society).

[B18-jintelligence-12-00038] Ho Eric, Jeon Minjeong (2023). Interaction map: A visualization tool for personalized learning based on assessment data. Psych.

[B19-jintelligence-12-00038] Hoff Peter D., Raftery Adrian E., Handcock Mark S. (2002). Latent space approaches to social network analysis. Journal of the American Statistical Association.

[B20-jintelligence-12-00038] Ishwaran Hemant, Rao J. Sunil (2005). Spike and slab variable selection: Frequentist and Bayesian strategies. The Annals of Statistics.

[B21-jintelligence-12-00038] Jeon Minjeong, Jin Ick Hoon, Schweinberger Michael, Baugh Samuel (2021). Mapping unobserved item–respondent interactions: A latent space item response model with interaction map. Psychometrika.

[B22-jintelligence-12-00038] Kang Inhan, Jeon Minjeong, Partchev Ivailo (2023). A latent space diffusion item response theory model to explore conditional dependence between responses and response times. Psychometrika.

[B23-jintelligence-12-00038] Kang Inhan, De Boeck Paul, Partchev Ivalio (2022a). A randomness perspective on intelligence processes. Intelligence.

[B24-jintelligence-12-00038] Kang Inhan, De Boeck Paul, Ratcliff Roger (2022b). Modeling conditional dependence of response accuracy and response time with the diffusion item response theory model. Psychometrika.

[B25-jintelligence-12-00038] Luo Jinwen, De Carolis Ludovica, Zeng Biao, Jeon Minjeong (2023). Bayesian estimation of latent space item response models with JAGS, Stan, and NIMBLE in R. Psych.

[B26-jintelligence-12-00038] Magis David, Béland Sébastien, Tuerlinckx Francis, De Boeck Paul (2010). A general framework and an R package for the detection of dichotomous differential item functioning. Behavioral Research Methods.

[B27-jintelligence-12-00038] Marsman Maarten, Borsboom Denny, Kruis Joost, Epskamp Sacha, van Bork Riet, Waldorp Lourens J., van der Maas Han L. J., Maris Gunter (2018). An introduction to network psychometrics: Relating ising network models to item response theory models. Multivariate Behavioral Research.

[B28-jintelligence-12-00038] Meng Xiang-Bin, Tao Jian, Chang Hua-Hua (2015). A conditional joint modeling approach for locally dependent item responses and response times. Journal of Educational Measurement.

[B29-jintelligence-12-00038] Mitchell Toby J., Beauchamp John J. (1988). Bayesian variable selection in linear regression. Journal of the American Statistical Association.

[B30-jintelligence-12-00038] Molenaar Dylan (2021). A flexible moderated factor analysis approach to test for measurement invariance across a continuous variable. Psychological Methods.

[B31-jintelligence-12-00038] Partchev Ivailo, De Boeck Paul (2012). Can fast and slow intelligence be differentiated?. Intelligence.

[B32-jintelligence-12-00038] Rasch Georg, Neyman Jerzy (1961). On general laws and meaning of measurement in psychology. Proceedings of the Fourth Berkeley Symposium on Mathematical Statistics and Probability, Volume 4: Contributions to Biology and Problems of Medicine.

[B33-jintelligence-12-00038] Roberts James S., Laughlin James E. (1996). A unidimensional item response model for unfolding responses from a graded disagree-agree response scale. Applied Psychological Measurement.

[B34-jintelligence-12-00038] Roberts James S., Donoghue John R., Laughlin James E. (2000). A general item response theory model for unfolding unidimensional polytomous responses. Applied Psychological Measurement.

[B35-jintelligence-12-00038] Smith Anna L., Asta Dena M., Calder Catherine A. (2019). The Geometry of Continuous Latent Space Models for Network Data. Statistical Science.

[B36-jintelligence-12-00038] Spearman Charles (1904). “General intelligence”, objectively determined and measured. The American Journal of Psychology.

[B37-jintelligence-12-00038] Stan Development Team (2024). Stan Modeling Language User’s Guide and Reference Manual Version 2.34. https://mc-stan.org/users/documentation/.

[B38-jintelligence-12-00038] Thissen David, Steinberg Lynne, Wainer Howard, Holland Paul W., Wainer Howard (1993). Detection of differential item functioning using the parameters of item response models. Differential Item Functioning.

[B39-jintelligence-12-00038] van der Linden Wim J. (2007). A hierarchical framework for modeling speed and accuracy on test items. Psychometrika.

[B40-jintelligence-12-00038] van der Linden Win J., Glas Cees A. W. (2010). Statistical tests of conditional independence between responses and/or response times on test items. Psychometrika.

[B41-jintelligence-12-00038] van der Maas Han L. J., Dolan Conor V., Grasman Raoul P. P. P., Wicherts Jelte M., Huizenga Hilde M., Raijmakers Maartje E. J. (2006). A dynamical model of general intelligence: The positive manifold of intelligence by mutualism. Psychological Review.

[B42-jintelligence-12-00038] van Rijn Peter W., Ali Usama S. (2017). A comparison of item response models for accuracy and speed of item responses with applications to adaptive testing. British Journal of Mathematical and Statistical Psychology.

[B43-jintelligence-12-00038] Wang Chun, Xu Gongjun (2015). A mixture hierarchical model for response times and response accuracy. British Journal of Mathematical and Statistical Psychology.

