# Supplementary material for: A Recent Development of a Network Approach to Assessment Data: Latent Space Item Response Modeling for Intelligence Studies
Source: J Intell. 2024 Mar 28;12(4):38. doi: 10.3390/jintelligence12040038 (PMC11050824; doi:10.3390/jintelligence12040038)
Supplement: Supplementary file 1 [file jintelligence-12-00038-s001.zip › jintelligence-2836290-supplementary.pdf]

SUPPLEMENTARY ONLINE MATERIAL

A Recent Development of a Network Approach to Assessment Data: Latent Space Item Response Modeling for Intelligence Studies

S1. Stan code for the *LSIRM* model

```
data {
  int<lower = 1> P;                 // number of persons
  int<lower = 1> I;                 // number of items
  int<lower = 1> N;                 // number of person-item pairs
  int<lower = 1, upper = P> pp[N];  // person index for the n-th obs
  int<lower = 1, upper = I> ii[N];  // item index for the n-th obs
  int<lower=0, upper=1> resp[N];    // response in the long format
  real mu[2];
  real kappa[2];
}
parameters {
  vector[P] std_theta;             // latent ability factors (standardized)
  vector[P] xi1;                   // latent person position 1
  vector[P] xi2;                   // latent person position 2
  vector[I] b;                     // item difficulty parameters
  vector[I] zt1;                   // latent item positions 1
  vector[I] zt2;                   // latent item positions 1
  real<lower = 0> omega_theta_sq;  // variance of latent ability factors
  real log_lambda;                 // distance tuning parameter
  real<lower = 0, upper = 1> pind; // PIP
}
transformed parameters{
  vector[P] theta;                 // latent ability factors (scaled)
  vector[N] dist;                  // distance terms
  real<lower = 0> omega_theta = sqrt(omega_theta_sq);
  real lambda;

  theta  = std_theta * omega_theta;
  lambda = exp(log_lambda);
```

```
  for (n in 1:N){
      dist[n] = sqrt((xi1[pp[n]] - zt1[ii[n]])^2 + (xi2[pp[n]] - zt2[ii[n]])^2);
  }
}
model {
  vector[2] lps;
  lps[1] = log(1-pind);
  lps[2] = log(pind);

  omega_theta_sq ~ cauchy(0, 25);
  std_theta     ~ std_normal();
  b ~ normal(0, 5);

  xi1 ~ std_normal();
  xi2 ~ std_normal();
  zt1 ~ std_normal();
  zt2 ~ std_normal();

  pind ~ beta(1,1);

  for(s in 1:2){
    lps[s] += normal_lpdf(log_lambda | mu[s], kappa[s]);
  }
  for (n in 1:N) {
    resp[n] ~ bernoulli_logit(theta[pp[n]] + b[ii[n]] - lambda * dist[n]);
  }
  target += log_sum_exp(lps);
}
```

S2. Convergence of Bayesian Chains

The histograms of potential scale reduction statistics ($\hat{R}$; Gelman, 1996; Gelman, Carlin, Stern, Dunson, & A. Vehtari, 2013) in Figure S1 and the trace plots of some randomly selected parameters shown in Figures S2-S3 present convergence assessment of the LSIRM model applied to our empirical examples. In general, the results do not imply any convergence issue. The values of $\hat{R}$ were all smaller than 1.01.

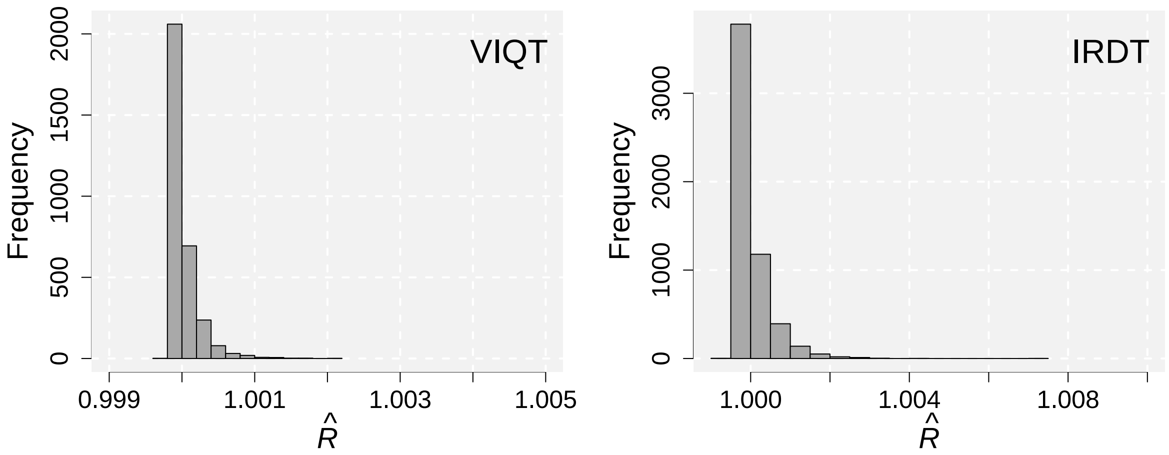

Figure S1. Histogram of Potential Scale Reduction Statistics ($\hat{R}$), from the LSIRM fit to the VIQT dataset (left) and the IRDT dataset (right).

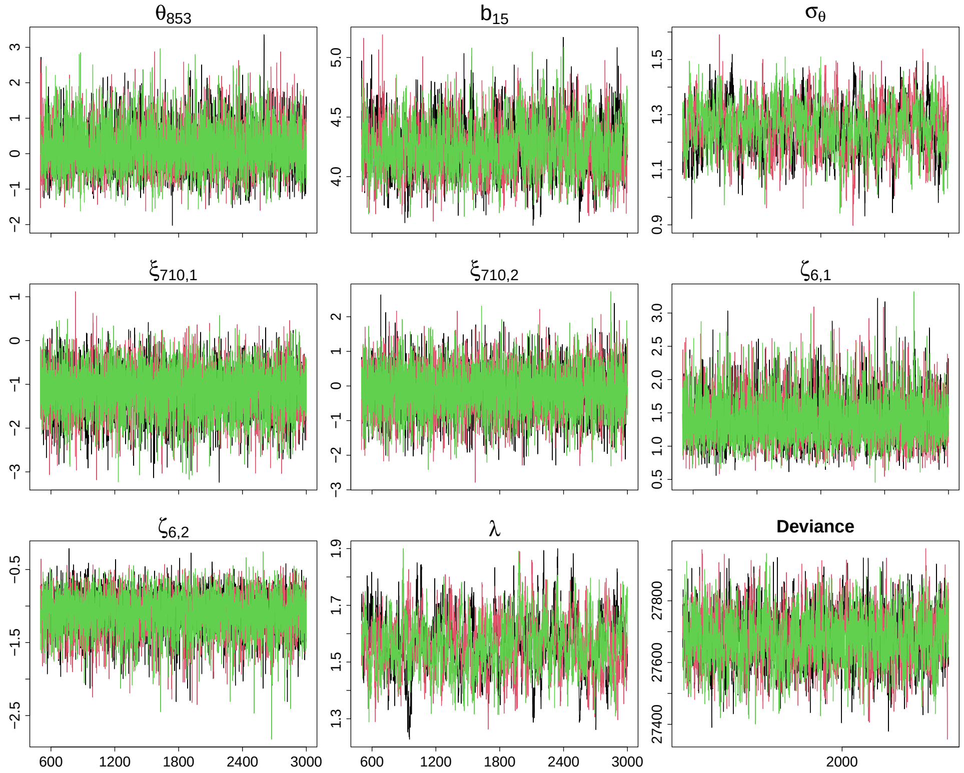

Figure S2. Trace Plots of Parameters for Randomly Selected Persons and Items in the VIQT Data Analysis.

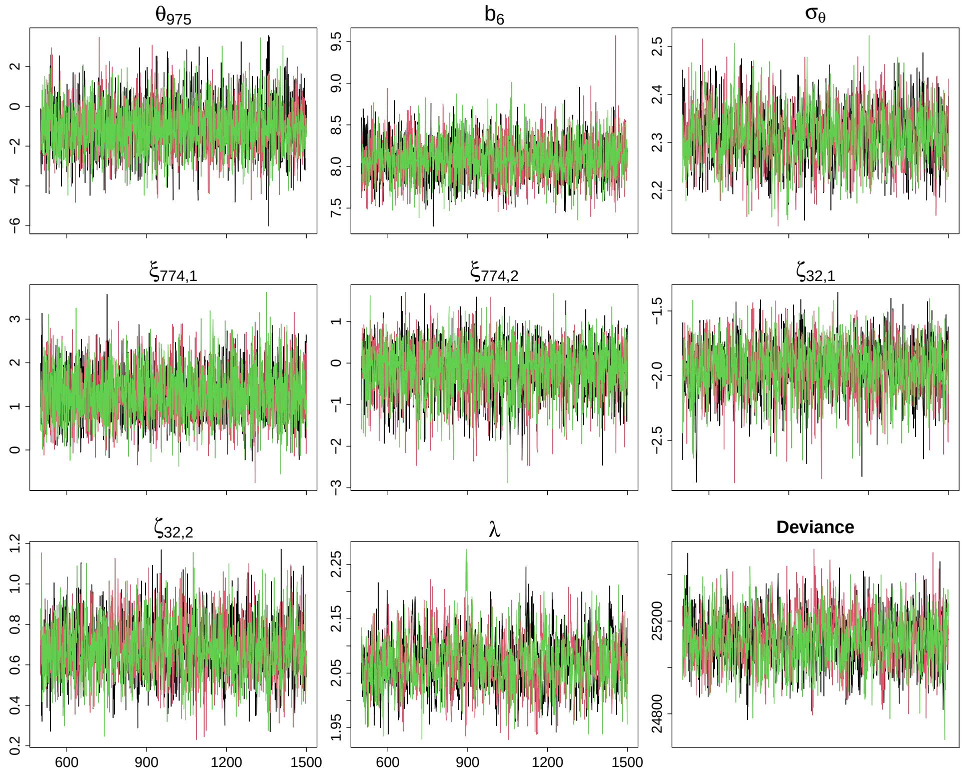

Figure S3. Trace Plots of Parameters for Randomly Selected Persons and Items in the IRDT Data Analysis.

References

Gelman, A. (1996). Inference and monitoring convergence. In W. R. Gilks, S. Richardson, & D. J. Spiegelhalter (Eds.), *Markov chain Monte Carlo in practice* (p. 131-143). CRC Press.

Gelman, A., Carlin, J. B., Stern, H. S., Dunson, D. B., & A. Vehtari, D. B. R. (2013). *Bayesian data analysis* (3rd ed.). CRC Press.
